# Extensive cross-reactive T cell epitopes across SARS-CoV-2 Omicron variant spikes with finite immune evasion mutations

**DOI:** 10.1186/s12967-025-07076-z

**Published:** 2025-09-30

**Authors:** Mengze Gan, Jinge Cao, Qi Ouyang, Xinyue Xu, Xingxing Wang, Peihang Dan, Yinlong Yao, Hui Fu, Xuanyu Yao, Xiaosong Lin, Qing Lei, Xionglin Fan

**Affiliations:** 1 Department of Pathogen Biology, School of Basic Medicine, Tongji Medical College and State Key Laboratory for Diagnosis and Treatment of Severe Zoonotic Infectious Diseases, Hubei Key Laboratory of Drug Target Research and Pharmacodynamic Evaluation, Huazhong University of Science and Technology, Wuhan, 430030 China; 2 Division of Nephrology, Tongji Hospital, Tongji Medical College, Huazhong University of Science and Technology, Wuhan, 430030 China

**Keywords:** SARS-CoV-2, Cross-T cell response, Spike, Mutation, Immune evasion, Omicron variant, T cell epitope

## Abstract

**Background:**

The impact of spike protein mutations on T cell responses, particularly in SARS-CoV-2 Omicron variants, remains incompletely elucidated.

**Methods:**

In this study, DNA vaccines encoding the spike protein of both the ancestral virus and Omicron variants were developed and administered in conjunction with a Th1-type adjuvant to BALB/c mice. Cross-reactive T cell responses to the spike proteins were assessed in the splenocytes of these mice using IFN-γ ELISPOT assays. Additionally, flow cytometry (FACS) was utilized to evaluate IFN-γ^+^ CD4^+^ and CD8^+^ T cell responses to various peptides covering the entire spike protein sequence.

**Results:**

Our study demonstrated that only a limited number of mice, along with a minor subset of their splenocytes vaccinated with the DNA vaccine targeting the original spike protein, exhibited weak cross-reactivity with Omicron variants. This observation underscores the differences in T cell epitopes between Omicron variants and the prototype spikes, indicating that at least 50 and 37 mutations in Omicron variants contribute to their evasion of CD4^+^ and CD8^+^ T cell responses, respectively. Conversely, DNA vaccines encoding spike proteins from Omicron variants successfully elicited strong cross-reactive T cell responses in the immunized mice. In particular, Vaccines targeting the BA.1, BA.5, XBB.1.5, and JN.1 variants demonstrated the most robust and comprehensive T cell immune responses among Omicron variants. This efficacy is attributed to the presence of less than eleven T cell immune evasion mutations in their spike proteins, alongside numerous mutations that enhance T cell responses.

**Conclusions:**

These findings underscore the imperative to update WHO emergency vaccine policies and contribute to the development of more effective vaccines and immunization strategies, to better control the infections caused by emerging Omicron variants.

**Supplementary Information:**

The online version contains supplementary material available at 10.1186/s12967-025-07076-z.

## Introduction

 The World Health Organization (WHO) announced that the COVID-19 pandemic has ended over two years [1]. According to recent data from sentinel surveillance of acute respiratory infectious diseases, the Chinese Center for Disease Control and Prevention (China CDC) recently reported that SARS-CoV-2 Omicron variants continue to circulate in China, accounting for 8.6% of severe hospitalizations [2]. Both vaccination [3, 4] and natural infection with SARS-CoV-2 [5] can elicit antiviral immune responses: neutralizing antibodies are crucial for preventing infection and limiting viral dissemination, while cellular immunity significantly reduces hospitalization rates, severe disease incidence, and mortality [6–8]. The spike protein not only constitutes the viral structures but also is a well-established key target antigen for the vaccines to elicit these protective immune responses [9]. All vaccines authorized for emergency use were developed derived from the SARS-CoV-2 prototype strain (PS) itself or its spike protein. Unfortunately, SARS-CoV-2 is characterized by a high mutation rate, particularly in its spike protein, which accumulates numerous mutations as the virus evolves. These mutations enhance the virus’s ability to evade neutralizing antibodies [10–12], thereby facilitating large-scale outbreaks of infections with these emerging variants. More importantly, our recent research reveals that the Omicron JN.1 variant with at least 50 mutations in its spike protein exhibits the strongest ability of the Omicron sublineage to escape neutralizing antibodies (manuscript in preparation). The viral evolution presents substantial challenges to current vaccines and immunization strategies [13, 14]. Consequently, there is an urgent need to develop more effective vaccines and immunization strategies to prevent and control the epidemics caused by emerging SARS-CoV-2 Omicron variant infections.

Besides neutralizing antibodies, the role of cellular immunity against SARS-CoV-2 early variants is also receiving increasing attention. Vaccination with various COVID-19 vaccines has been demonstrated to elicit spike protein-specific CD4^+^ and CD8^+^ T cell responses, which secrete interferon-gamma (IFN-γ) and tumor necrosis factor-alpha (TNF-α) [15–17]. These immune responses were confirmed to play important roles in preventing severe clinical outcomes against SARS-CoV-2 PS or its early variants [18, 19]. Interestingly, T cells from both naturally infected and vaccinated individuals are capable of recognizing the spike protein of early SARS-CoV-2 variants [20], and these early variants are unable to completely evade the host’s pre-established cellular immune responses [7, 8, 21, 22]. However, the impact of spike mutations on cellular immunity remains complex. Other studies have suggested that mutations in the spike protein may compromise CD4^+^ T cell response [23], impair pre-existing T cell responses [24], or damage the immune surveillance function of CD8^+^ T cells [25]. Furthermore, some studies have indicated that mutations in the Omicron BA.1 [26] and BA.4/5 [27] spikes also contribute to the modulation of cellular immune responses, facilitating viral immune evasion following vaccination or infection. A comprehensive analysis of cross-T cell responses among the PS, early variants such as Alpha, Beta, Gamma, and Delta, and Omicron BA.1 spike proteins were demonstrated by DNA vaccination in mice, and indicated that these early variants significantly evade the cellular immune response elicited by the DNA vaccine targeting the PS spike protein, and only Delta and Omicron BA.1 variants induce broad cross-reactive T cell responses to other spikes [28]. Notably, the ability of T cell immune evasion has emerged as a critical factor influencing the severity and mortality associated with infections by newly emerged SARS-CoV-2 variants, particularly the Omicron sublineages [29]. Especially importantly, due to the high immunization coverage with the inactivated SARS-CoV-2 PS vaccine and the high breakthrough infection rate of during the outbreak of Omicron BA.5/BF.7 variant immediately following the lifting of non-pharmacological interventions, the Chinese population has developed hybrid immunity. The key question is whether the current high rate of severe hospitalization in COVID-19 is due to the fact that the prevailing Omicron variants escaped the pre-established cellular immunity? In other words, the impact of the mutations, especially in the spike protein of Omicron variants, on T cell-mediated immune responses, is still not fully understood. To achieve this objective, the present study aims to develop DNA vaccines utilizing the coding sequences of the spike protein of the SARS-CoV-2 PS and the predominant variants of the Omicron sublineages. Following the immunization of BALB/c mice, cross-T cell responses to the spike protein of the PS and Omicron variants were assessed and compared using the IFN-γ enzyme-linked immunospot assay (ELISPOT). Hypothetical T cell epitopes covering the full-length sequence of these spike proteins were commercially synthesized. Fluorescence Activated Cell Sorting (FACS) and intracellular cytokine staining (ICS) were employed to detect peptide-specific CD4^+^ or CD8^+^ T cells that secret IFN-γ, thereby constructing a landscape of peptide-specific T cell responses and elucidating the effect of each mutation on T cell responses. Our findings are critical to update vaccines or immunization strategies to copy with the prevalence of current or future viral variants.

## Methods

### Plasmid construction and identification

Signal peptide from human IgE sequence was utilized, and the coding sequences for spike proteins of PS, Omicron predominant variants such as BA.1, BA.5, XBB.1.5, CH.1.1, EG.5.1, and JN.1 were synthesized commercially and cloned into the pVAX-1 vector. This process yielded recombinant eukaryotic expression plasmids pVAX-PS, pVAX-BA.1, pVAX-BA.5, pVAX-XBB.1.5, pVAX-CH.1.1, pVAX-EG.5.1, and pVAX-JN.1, as detailed in our previous study [28].

### Immunization of mice with DNA vaccine

DNA vaccine was prepared as previously described [30]. In brief, the recombinant plasmid was mixed with 100 μL (100 μg) of DNA and 100 μL of the DM adjuvant with vortexed every 30 min. Groups of twenty female specific-pathogen-free (SPF) BALB/c mice, aged 6–8 weeks, were immunized twice by an intramuscular injection of 200 μL of the DNA/DM vaccine per mouse, with a 3-week interval between doses. Six weeks post-final immunization, spleens were collected for further analysis. The research protocols were reviewed and approved by the Committee on the Ethics of Animal Experiments of Tongji Medical College (IACUC Number 4516).

### Detection of cross-T cell response to the spike protein by ELISPOT

Splenocytes were prepared from each mouse. Mouse IFN-γ ELISPOT kits (BD Biosciences, Cat. #551083, USA) were used to detect cross-T cell responses to the spike protein of the PS and Omicron variants (Sino Biological), as previously described in detail [28]. If the number of spots in the negative control wells is ≤ 5, then the experimental wells minus the negative control wells > 5 are defined as positive. If the number of spots in the negative control wells is greater than 5, then a ratio of experimental wells to negative control Wells of ≥ 2 is defined as positive.

### Detection of peptide‐specific IFN‐γ^+^ CD4^+^ and IFN‐γ^+^ CD8^+^ T cell responses

Epitope peptides, encompassing the entire length of the spike protein, were designed based on the PS spike protein. These peptides consisted of 16 amino acids, with an overlap of 8 amino acids at both the N- and C-termini. Sixteen-mer peptides were selected to balance structural compatibility with MHC class I/II binding grooves, computational prediction efficacy, and biological relevance to APC processing dynamics. Another 153 peptides with the mutation site corresponding to the PS peptides were synthesized, with each incorporating mutation sites specific to Omicron variants such as BA.1, BA.2, BA.4, BA.5, BF.7, XBB.1.5, BQ.1, CH.1.1, EG.5.1, HV.1, and JN.1. FACS and ICS techniques were used to detect peptide‐specific IFN‐γ^+^ CD4^+^ and IFN-γ ^+^ CD8^+^ T cell responses, as detailed in our previous research [28]. For data analysis, a minimum of 5 × 10^4^ cells were collected per sample. The results were expressed as the percentage of cells in the experimental well minus the percentage of cells in the negative control well, with negative values standardized to zero. Positive standard judgment is defined as follows: when the result of the positive control well is satisfactory, the ratio of the experimental well to the negative control well is ≥ 2, and the simultaneous detection value is ≥ 0.01, it is classified as positive. The criteria for assessing peptide escape of Omicron variants from the PS are as follows: if the PS peptide response is lower than the positive threshold, the variant strain peptide response exceeds the positive threshold, and the T-cell response to the variant strain peptide is significantly greater than that to the PS peptide, it is considered T-cell immune escape. For identifying peptides that have escaped from other Omicron variants compared to the BA.5 variant, the criteria are: if the BA.5 variant peptide response exceeds the positive threshold, the peptides of other Omicron variants fall below this threshold, and the T-cell response to the BA.5 variant peptide is significantly greater than that to the peptides of other Omicron variants, it is classified as T-cell immune escape.

### Statistical analysis

Statistical difference analysis and data visualization were performed using Prism 8 software (GraphPad). The cross-T-cell response was evaluated using one-way ANOVA, while comparisons of peptide-specific T-cell responses was assessed using multiple t-tests. Statistical difference was set when a P value less than 0.05. Significance levels were denoted as follows: *P ≤ 0.05, **P ≤ 0.01, ***P ≤ 0.001.

## Results

### Omicron variant spikes demonstrate substantial cross-T cell responses with one another and PS

**Fig. 1:**
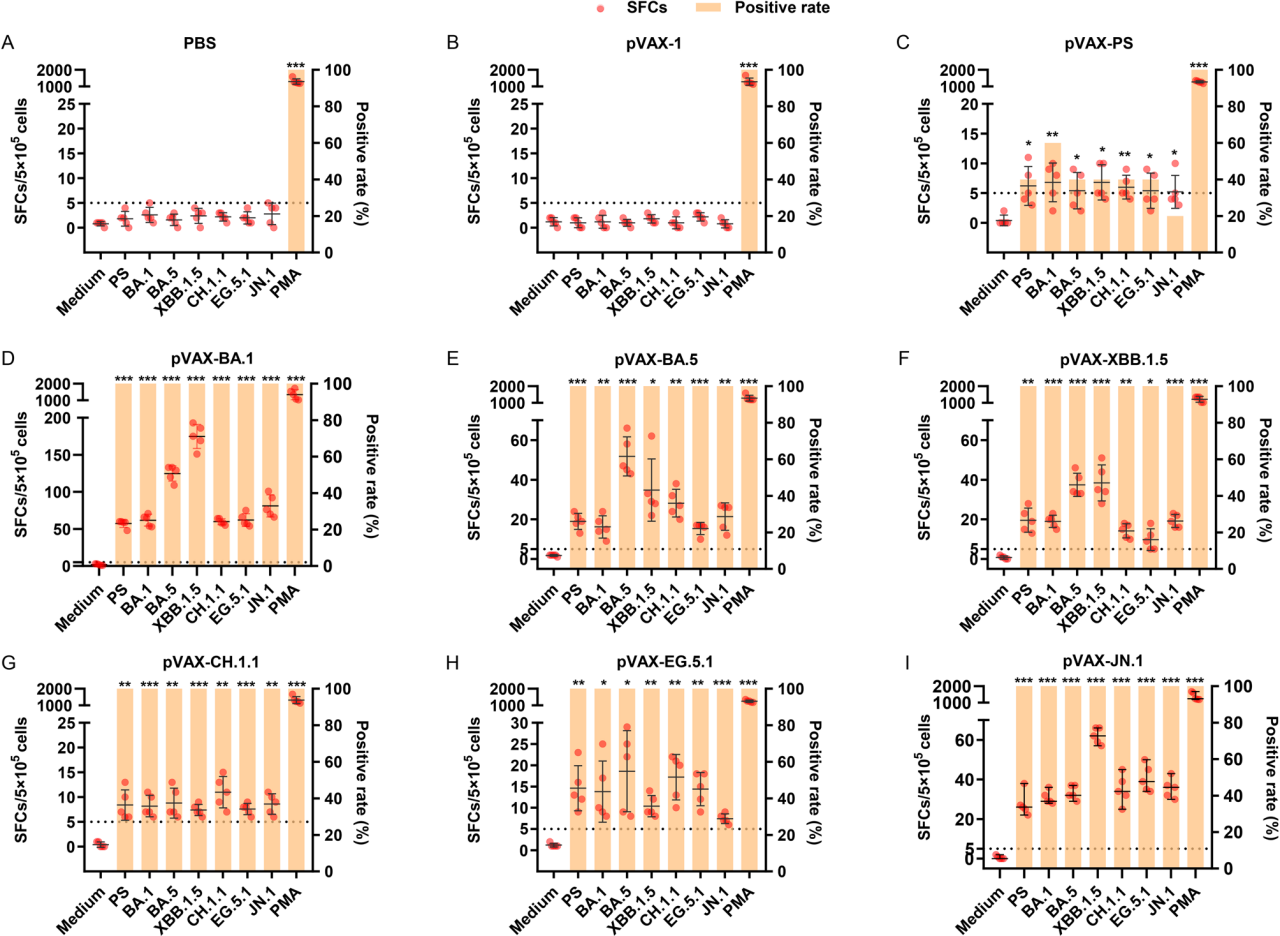
Comparison of cross-T cell responses to the spike protein among the SARS-CoV-2 prototype strain and Omicron variants (n = 5). Splenocytes from BALB/c mice immunized with the prototype strain (PS), and Omicron BA.1, BA.5, XBB.1.5, CH.1.1, EG.5.1, and JN.1 DNA vaccines were collected. IFN-γ ELISPOT assay was employed to detect and compare the number of spike protein-specific IFN-γ secreting T cells across PS and the different Omicron variants. The results were expressed as the median ± interquartile range of spot forming cells (SFC). (**A**) PBS control; (**B**) pVAX-1; (**C**) pVAX-PS; (**D**) pVAX-BA.1; (**E**) pVAX-BA.5; (**F**) pVAX-XBB.1.5; (**G**) pVAX-CH.1.1; (**H**) pVAX-EG.5.1; (**I**)pVAX-JN.1. Red dots represent the number of SFC, while yellow columns represent the positive rate of T cell responses. *P* ≤ 0.05, *P* ≤ 0.01, *P* ≤ 0.001

Cross-T cell responses are crucial for assessing the protective efficacy of vaccines against viral variants. To investigate the cross-T cell responses among different Omicron variants, splenocytes from mice immunized with PS, and Omicron BA.1, BA.5, XBB.1.5, CH.1.1, EG.5.1, and JN.1 DNA vaccines were collected. The IFN-γ ELISPOT assay was employed to detect and compare the number of spike protein-specific IFN-γ secreting T cells across PS and the different Omicron variants. As expected, none of the culture medium controls in any group elicited specific T cell responses, whereas positive stimulation of PMA resulted in elevated T cell responses. Additionally, neither the PBS group nor the pVAX-1 group showed specific T cell responses to any of the spike proteins (Fig. 1A-B).

Interestingly, 40% (2 out of 5) of mice immunized with pVAX-PS DNA exhibited cross-reactive T cell responses against the spike proteins of PS, BA.5, XBB.1.5, CH.1.1, and EG.5.1. Notably, 60% (3 out of 6) and 20% (1 out of 5) of mice elicited the cross-reactive T cell response to BA.1 and JN.1 spike proteins, respectively. Even so, all of these immune responses were weak because the mean SFC in each response was relatively low (Fig. 1C). These results indicate that the T cell response induced by PS spike protein can weakly recognize Omicron variant spike proteins. Remarkably, all mice (100%, 5 out of 5) in each Omicron variant DNA vaccinated group demonstrated cross-reactive T cell responses to other Omicron variants and the PS, with relatively high mean SFC in each response. Of all groups, BA.1 spike DNA vaccination induced the most potent and broad cross-reactive T cell responses to other spikes, followed sequentially by BA.5, XBB.1.5, and JN.1 spikes (Fig. 1D-I).

### Omicron variants exhibit cross-T cell responses to SARS-CoV-2 PS spike peptides within the RBD region

**Fig. 2:**
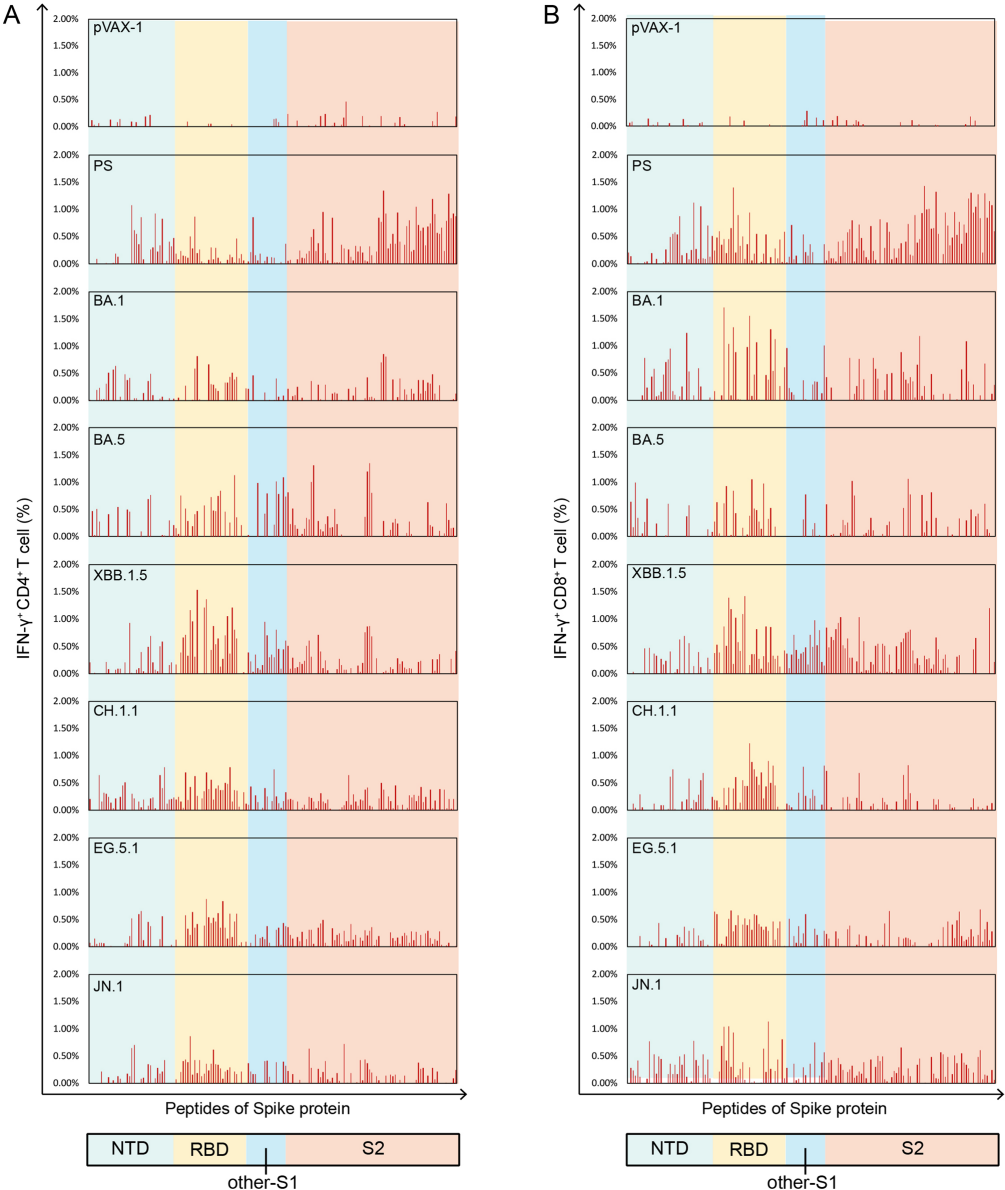
Landscapes of T cell responses to SARS-CoV-2 prototype strain spike protein peptides in different DNA vaccinated mice (n = 3). BALB/c mice were immunized with the prototype strain (PS), and Omicron BA.1, BA.5, XBB.1.5, CH.1.1, EG.5.1, and JN.1 DNA vaccines, respectively. 158 peptides spanning the full length of the PS spike protein were employed to stimulate splenocytes from all DNA vaccinated mice, respectively. FACS and ICS were utilized to detect peptide-specific IFN-γ^+^ CD4^+^ and IFN-γ^+^ CD8^+^ T cell responses. (**A**) A comparative landscape of peptide-specific IFN-γ^+^ CD4^+^ T cell responses between pVAX-1 control and different DNA vaccinated groups. (**B**) A comparative landscape of peptide-specific IFN-γ^+^ CD8^+^ T cell responses between pVAX-1 control and different DNA vaccinated groups

To construct the landscapes of spike protein peptide-specific T cell responses, 158 peptides spanning the full length of the PS spike protein were employed to stimulate splenocytes from all DNA vaccinated mice. Given that ICS enables dead-cell exclusion, distinguishes contributions from different T-cell subsets, and requires lower cell input requirements, we selected ICS over ELISPOT for subsequent investigations. FACS and ICS were utilized to detect peptide-specific IFN-γ^+^ CD4^+^ and IFN-γ^+^ CD8^+^ T cell responses, and the landscape of peptide-specific T cell responses was successfully constructed (Fig. 2). As expected, stimulation of splenocytes from pVAX-1 control mice with PS peptides elicited negligible T cell responses. In the pVAX-PS group, the threshold for CD4^+^ T cell activation was determined to be 0.14%. Upon stimulation with PS peptides, 54.4% (86 out of 158) of the peptides in the pVAX-PS group induced IFN-γ^+^ CD4^+^ T cell responses. In comparison, the pVAX-BA.1, pVAX-BA.5, pVAX-XBB.1.5, pVAX-CH.1.1, pVAX-EG.5.1, and pVAX-JN.1 groups exhibited IFN-γ^+^ CD4^+^ T cell responses in 37.3% (59/158), 36.7% (58/158), 44.9% (71/158), 36.1% (57/158), 51.3% (81/158), and 44.3% (70/158) of the PS peptides, respectively (Fig. 2A).

The cut-off value for CD8^+^ T cell activation was established at 0.31% in the pVAX-PS group. Stimulation of splenocytes from pVAX-PS mice with the PS peptides, 47.5% (75/158) of the PS peptides resulted in IFN-γ^+^ CD8^+^ T cell responses. In the pVAX-BA.1, pVAX-BA.5, pVAX-XBB.1.5, pVAX-CH.1.1, pVAX-EG.5.1, and pVAX-JN.1 groups, IFN-γ^+^ CD8^+^ T cell responses were observed in 36.7% (58/158), 28.5% (45/158), 48.7% (77/158), 33.5% (53/158), 33.5% (53/158), and 34.2% (54/158) of the PS peptides, respectively. Based on the comparative landscape analysis, Omicron variants exhibited cross-T cell responses to the PS peptides widely distributed in the N-terminal domain (NTD), receptor-binding domain (RBD), other S1, and S2 regions, with the highest concentration observed in the RBD region of the PS spike protein (Fig. 2B).

### Omicron spike proteins have at least 50 T cell immune evasion mutations compared to the PS

To investigate the impact of mutations on T cell epitopes across different Omicron variants, this study employed 153 mutant peptides encompassing all mutation sites on the spike protein of Omicron variants. Splenocytes from mice in the pVAX-BA.1, pVAX-BA.5, pVAX-XBB.1.5, pVAX-CH.1.1, pVAX-EG.5.1, and pVAX-JN.1 groups were stimulated with both PS and respective mutant peptides, and peptide-specific IFN-γ^+^ CD4^+^ and IFN-γ^+^ CD8^+^ T cell responses were assessed, respectively.

**Fig. 3:**
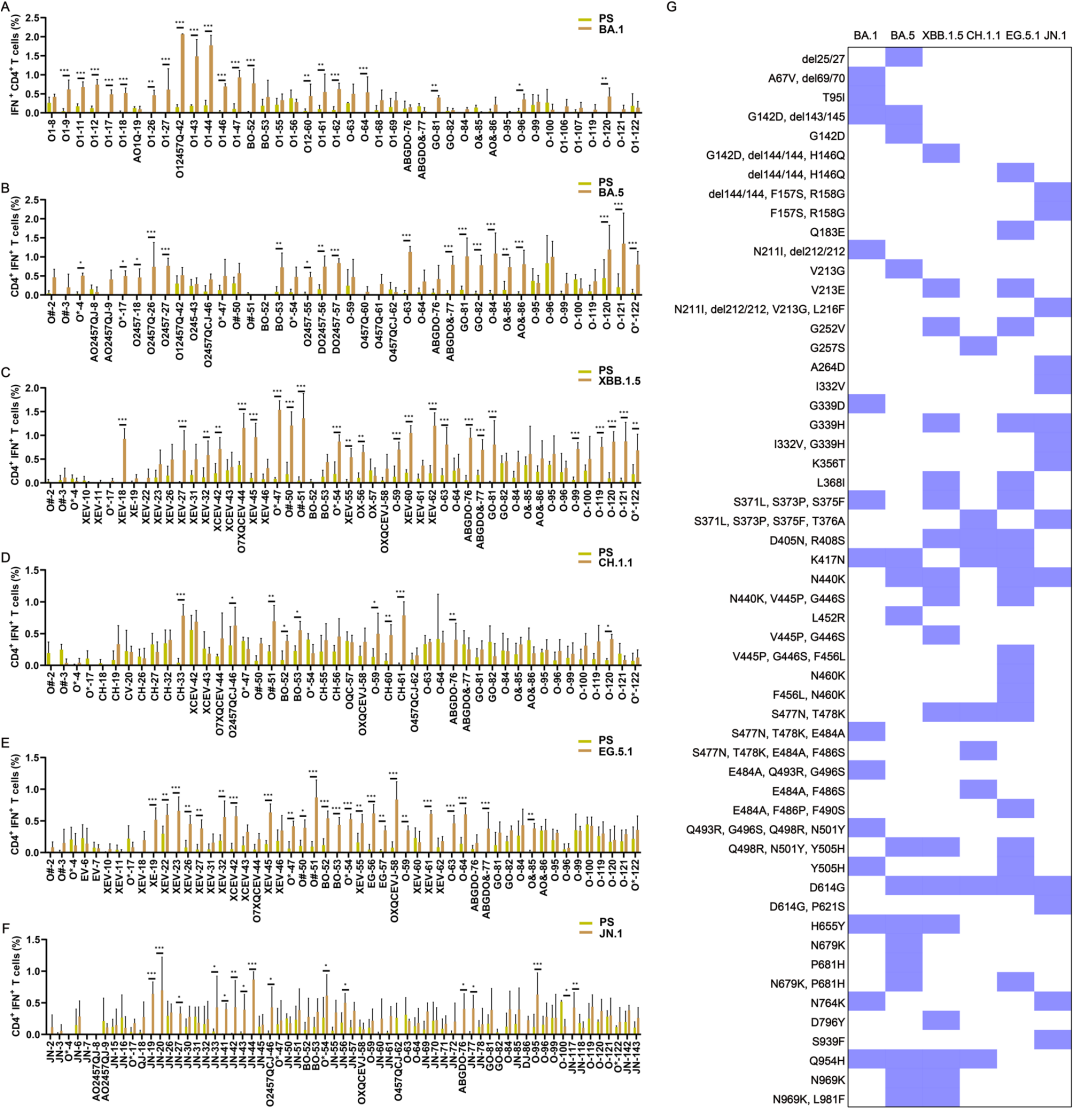
CD4^+^ T cell immune evasion mutations in Omicron spike proteins compared to the PS (n = 3). BALB/c mice were immunized with the prototype strain (PS), and Omicron BA.1, BA.5, XBB.1.5, CH.1.1, EG.5.1, and JN.1 DNA vaccines, respectively. were employed to stimulate splenocytes from all DNA vaccinated mice, respectively. 153 peptides with the mutation site corresponding to the PS peptides in Omicron variants such as BA.1, BA.2, BA.4, BA.5, BF.7, XBB.1.5, BQ.1, CH.1.1, EG.5.1, HV.1, and JN.1, were generated. FACS and ICS were utilized to detect these peptide-specific IFN-γ^+^ CD4^+^ T cell responses, and 158 peptides spanning the full length of the PS spike protein were used as controls. Comparison of IFN-γ^+^ CD4^+^ T cell responses to the PS peptides and BA.1 mutant peptides (**A**); BA.5 mutant peptides (**B**); XBB.1.5 mutant peptides (**C**); CH.1.1 mutant peptides (**D**); EG.5.1 mutant peptides (**E**); and JN.1 mutant peptides (**F**). (**G**) All CD4.^+^ T cell immune evasion mutations in Omicron spike proteins. The blue color represents T cell immune evasion mutations, while the white color represents non-escape mutations. *P* ≤ 0.05, *P* ≤ 0.01, *P* ≤ 0.001

In the pVAX-BA.1 group, 47.6% (20 out of 42) of the BA.1 mutant peptides elicited higher levels of IFN-γ^+^ CD4^+^ T cell responses compared to the corresponding PS peptides, and most of them fulfill the criteria for immune evasion, except the peptides corresponding to the G339D-R346K and R346K mutant sites. At least 14 mutant sites were identified for T cell immune evasion in the BA.1 spike protein, including A67V-del69/70, T95I, G142D-del143/145, N211I-del212/212, G339D, S371L-S373P-S375F, K417N, S477N-T478K-E484A, E484A-Q493R-G496S, Q493R-G496S-Q498R-N501Y, Y505H, H655Y, N764K, Q954H (Fig. 3A). Based on the same techniques, we found that 45.2% (19 out of 42) of BA.5 mutant peptides (Fig. 3B), 48.0% (24 out of 50) of XBB.1.5 mutant peptides (Fig. 3C), 21.7% (10 out of 46) of the CH.1.1 mutant peptides (Fig. 3D), 46.2% (24 out of 52) of the EG.5.1 mutant peptides (Fig. 3E), and 23.1% (15 out of 65) of the JN.1 mutant peptides (Fig. 3F), elicited higher levels of IFN-γ^+^ CD4^+^ T cell responses than the corresponding PS peptides in each DNA vaccinated mice, respectively. The peptides corresponding to the mutant sites such as G252V, R346T, L368I, T376A, V445P/H, G446S, N450D, L452W, L455S, F486P, F490S, N764K, N969K, and L981F, were excluded, because they did not meet the positive criteria for immune evasion. A total of at least 50 mutant sites were discovered for CD4^+^ T cell immune evasion in each spike of the Omicron variants and summarized as shown in Figs. 3G. Among these immune escaping sites, 20 mutations presented simultaneously in several Omicron variants, such as G142D, del 143–145, V213G/E, G339D/H, S371L, S373P, S375F, D405N, R408S, K417N, N440K, S477N, T478K, E484A, Q498R, N501Y, Y505H, D614G, H655Y, and Q954H.

**Fig. 4:**
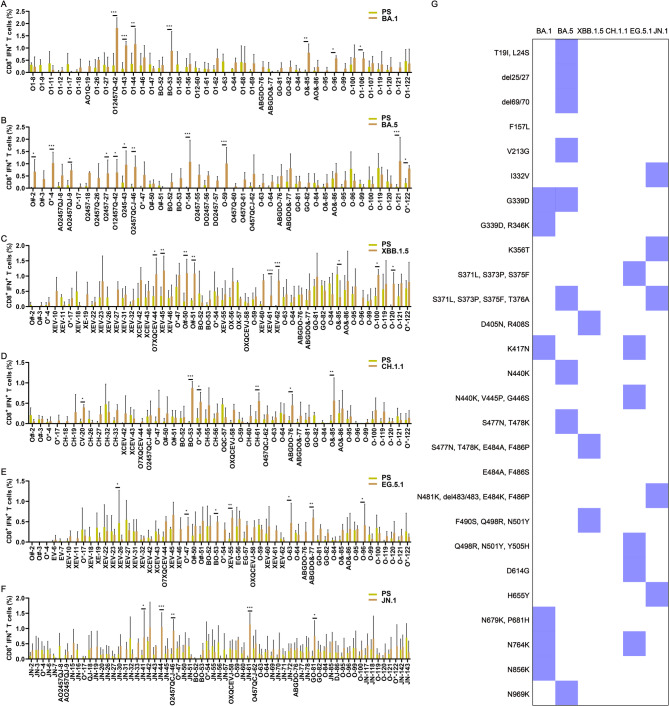
CD8^+^ T cell immune evasion mutations in Omicron spike proteins compared to the PS (n = 3). BALB/c mice were immunized with the prototype strain (PS), and Omicron BA.1, BA.5, XBB.1.5, CH.1.1, EG.5.1, and JN.1 DNA vaccines, respectively. FACS and ICS were utilized to detect peptide-specific IFN-γ^+^ CD8^+^ T cell responses. Comparison of IFN-γ^+^ CD8^+^ T cell responses to the PS peptides and BA.1 mutant peptides (**A**); BA.5 mutant peptides (**B**); XBB.1.5 mutant peptides (**C**); CH.1.1 mutant peptides (**D**); EG.5.1 mutant peptides (**E**); and JN.1 mutant peptides (**F**). (**G**) All CD8.^+^ T cell immune evasion mutations in Omicron spike proteins. The blue color represents T cell immune evasion mutations, while the white color represents non-escape mutations. *P* ≤ 0.05, *P* ≤ 0.01, *P* ≤ 0.001

Correspondingly, 16.7% (7 out of 42) of the BA.1 mutant peptides (Fig. 4A), 26.2% (11 out of 42) of BA.5 mutant peptides (Fig. 4B), 16.0% (8 out of 50) of XBB.1.5 mutant peptides (Fig. 4C), 13.0% (6 out of 46) of the CH.1.1 mutant peptides (Fig. 4D), 16.7% (7 out of 52) of the EG.5.1 mutant peptides (Fig. 4E), and 7.7% (5 out of 65) of the JN.1 mutant peptides (Fig. 4F), elicited a higher IFN-γ^+^ CD8^+^ T cell response compared to the respective PS peptides. The peptides corresponding to the mutate sites such as R346T, L368I, D796Y, Q954H, and L981F, were excluded, which did not satisfy the positive criteria for immune escape. A total of at least 37 mutant sites were discovered for CD8^+^ T cell immune evasion in each spike of the Omicron variants and summarized as shown in Figs. 4G. Among these immune escaping sites, only a few mutations presented simultaneously in several Omicron variants, such as G339D, S371L, S373P, S375F, K417N, N440K, and N764K.

### Other Omicron variant spike proteins have a few T cell immune evasion mutations compared to the BA.5

**Fig. 5:**
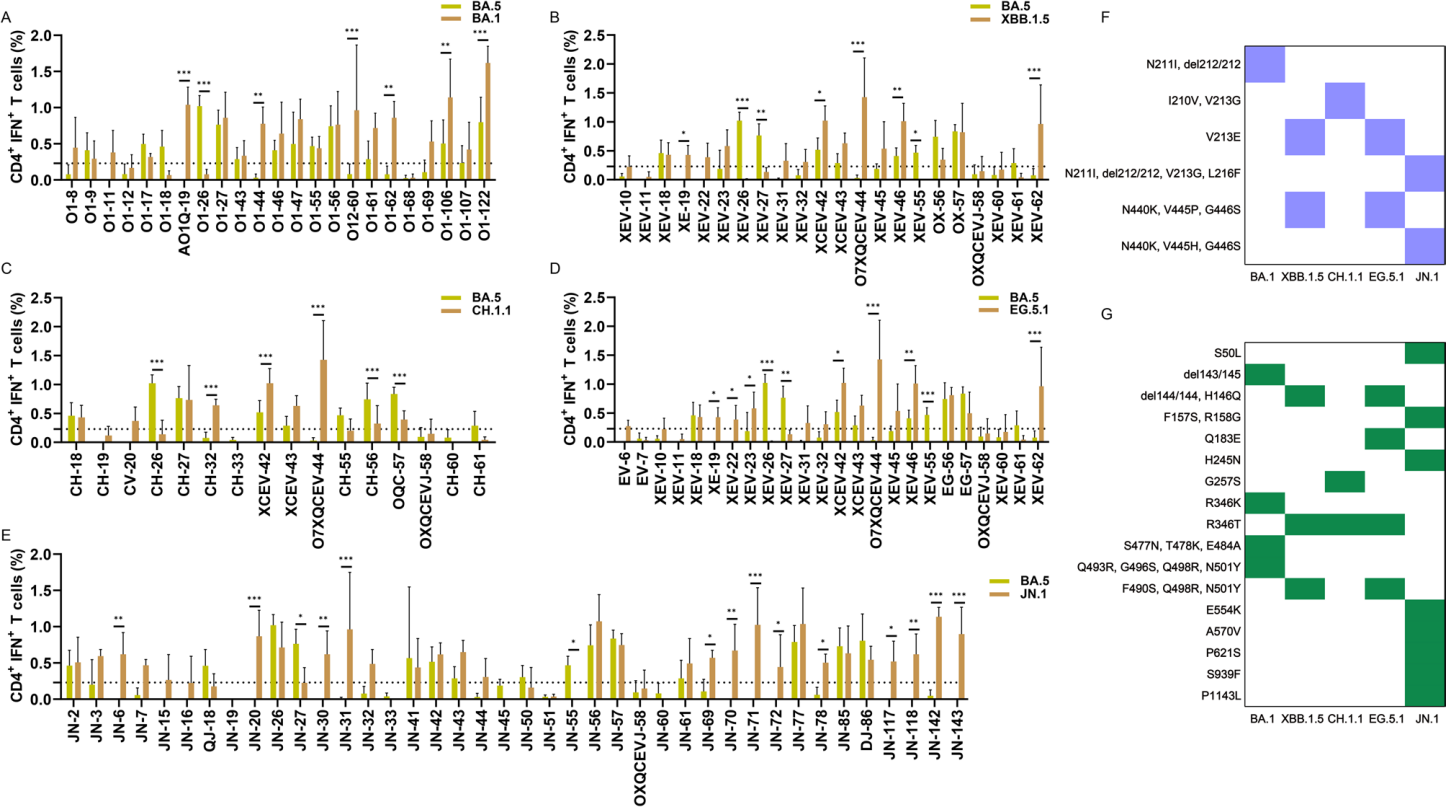
CD4^+^ T cell immune evasion mutations in other Omicron spike proteins compared to the BA.5 (n = 3). BALB/c mice were immunized with Omicron BA.1, BA.5, XBB.1.5, CH.1.1, EG.5.1, and JN.1 DNA vaccines, respectively. FACS and ICS were utilized to detect peptide-specific IFN-γ^+^ CD4^+^ T cell responses. Comparison of IFN-γ^+^ CD4^+^ T cell responses to the BA.5 mutant peptides and BA.1 mutant peptides (**A**); XBB.1.5 mutant peptides (**B**); CH.1.1 mutant peptides (**C**); EG.5.1 mutant peptides (**D**); and JN.1 mutant peptides (**E**); (**F**) All CD4.^+^ T cell immune evasion mutations in other Omicron spike proteins except the BA.5. The blue color represents T cell immune evasion mutations, while the white color represents non-escape mutations. (**G**) The mutation sites of other Omicron variants that enhance T cell responses. compared with the BA.5 variant. The green color represents T cell immune enhance mutations, while the white color represents non-enhance mutations. *P* ≤ 0.05, *P* ≤ 0.01, *P* ≤ 0.001

Following the pandemic of the Omicron BA.5/BF.7 variant, the Chinese population developed hybrid immunity. To assess the impact of the mutations in the spikes of other Omicron variants on the pre-existing T cell responses after infection with the Omicron BA.5/BF.7, mutant peptides from Omicron variants such as BA.1, XBB.1.5, CH.1.1, EG.5.1, and JN.1 were respectively employed to stimulate splenocytes of the pVAX-BA.5 vaccinated mice to evaluate specific IFN-γ^+^ CD4^+^ and CD8^+^ T cell responses. BA.5 mutant peptides were used as controls. 26.1% (6 out of 23) of the BA.1 mutated peptides (Fig. 5A), 22.7% (5/22) of XBB.1.5 mutated peptides (Fig. 5B),18.8% (3 out of 16) of the CH.1.1 mutated peptides (Fig. 5C), 29.2% (7 out of 24) of the EG.5.1 mutant peptides (Fig. 5D), 32.5% (13 out of 40) of the JN.1 mutated peptides (Fig. 5E), elicited a higher IFN-γ^+^ CD4^+^ T cell response compared to the BA.5 mutant peptides. The corresponding peptides with these mutant sites attributed to cross-reactive CD4^+^ T cell responses among Omicron variant spikes. Conversely, 4.3% (1 out of 23) of the BA.1 mutant peptides, 13.6% (3/22) of XBB.1.5 mutant peptides, 18.8% (3 out of 16) of CH.1.1 mutated peptides, 12.5% (3 out of 24) of the EG.5.1 mutant peptides, and 5.0% (2 out of 40) of the JN.1 mutated peptides induced a lower IFN-γ^+^ CD4^+^ T cell response compared to the BA.5 mutated peptides, meeting the criteria for T cell immune evasion. Only eight mutations such as I210V, N211I, del212/212, V213E/G, L216F, N440K, V445P/H, and G446S were discovered to evade the CD4^+^ T cell response associated with BA.5 (Fig. 5F). The mutate sites that enhance the CD4^+^ T cell responses in Omicron variant spikes were summarized as Fig. 5G.

**Fig. 6:**
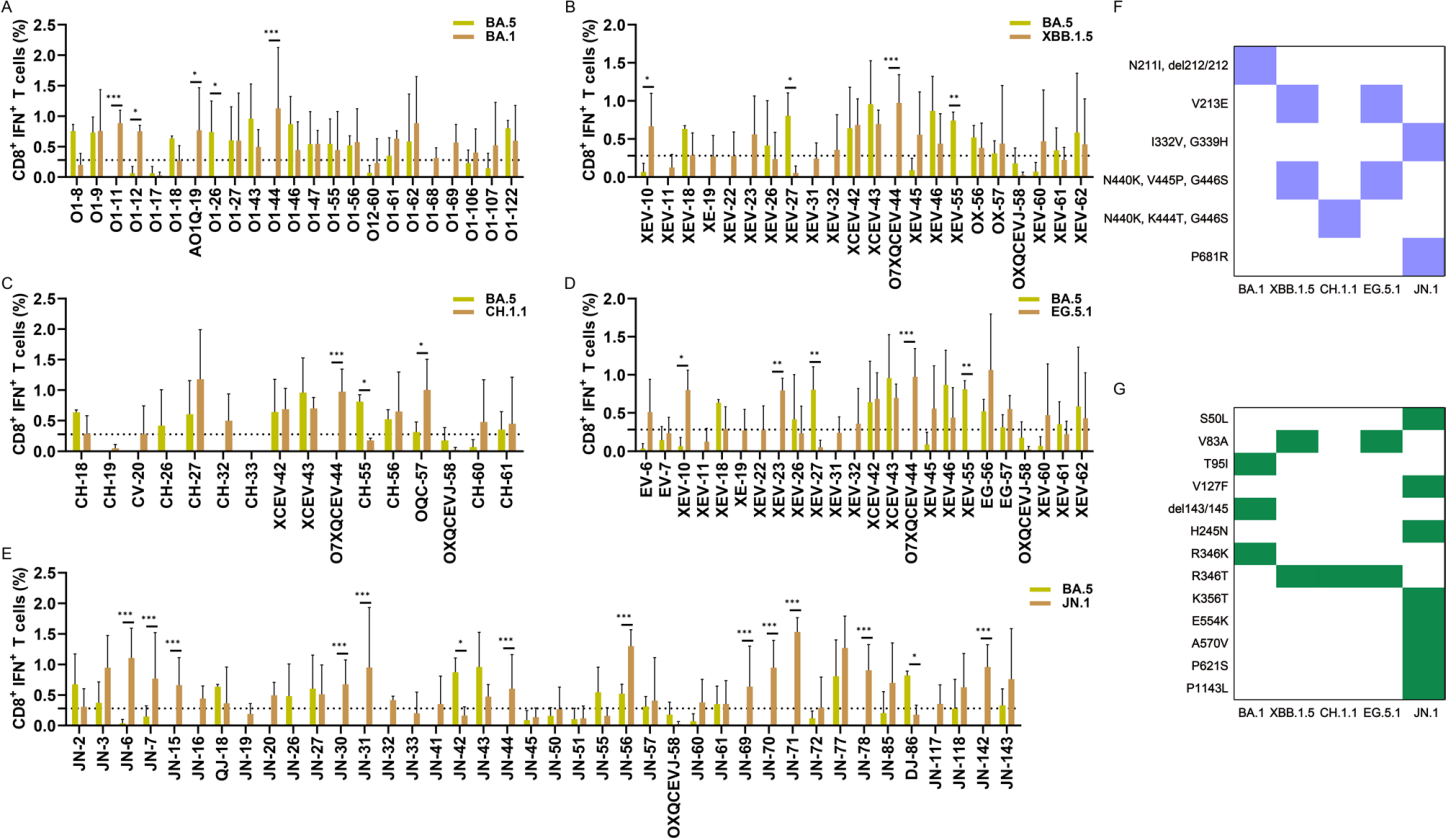
CD8^+^ T cell immune evasion mutations in other Omicron spike proteins compared to the BA.5 (n = 3). BALB/c mice were immunized with Omicron BA.1, BA.5, XBB.1.5, CH.1.1, EG.5.1, and JN.1 DNA vaccines, respectively. FACS and ICS were utilized to detect peptide-specific IFN-γ^+^ CD8^+^ T cell responses. Comparison of IFN-γ^+^ CD8^+^ T cell responses to the BA.5 mutant peptides and BA.1 mutant peptides (**A**); XBB.1.5 mutant peptides (**B**); CH.1.1 mutant peptides (**C**); EG.5.1 mutant peptides(**D**); and JN.1 mutant peptides (**E**); (**F**) All CD8.^+^ T cell immune evasion mutations in other Omicron spike proteins except the BA.5. The blue color represents T cell immune evasion mutations, while the white color represents non-escape mutations. (**G**) The mutation sites of other Omicron variants that enhance T cell responses. compared with the BA.5 variant. The green color represents T cell immune enhance mutations, while the white color represents non-enhance mutations. *P* ≤ 0.05, *P* ≤ 0.01, *P* ≤ 0.001

Similarly, 17.4% (4 out of 23) of BA.1 mutant peptides (Fig. 6A), 9.1% (2/22) of XBB.1.5 mutant peptides (Fig. 6B), 12.5% (2 out of 16) of CH.1.1 mutated peptides (Fig. 6C), 12.5% (3 out of 24) of the EG.5.1 mutant peptides (Fig. 6D), and 30.0% (12 out of 40) of the JN.1 mutated peptides (Fig. 6E), triggered a stronger IFN-γ^+^ CD8^+^ T cell response compared to the BA.5 mutant peptides, indicating an enhancement of the CD8^+^ T cell response against BA.5. The corresponding peptides with these mutant sites attributed to cross-reactive CD8^+^ T cell responses among Omicron variant spikes. In contrast, 4.3% (1/23) of the BA.1 mutant peptides, 9.1% (2/22) of XBB.1.5 mutant peptides, 6.25% (1 out of 16) of CH.1.1 mutated peptides, 8.33% (2 out of 24) of the EG.5.1 mutant peptides, and 5.0% (2 out of 40) of the JN.1 mutated peptides, resulted in a reduced IFN-γ^+^ CD8^+^ T cell response relative to the BA.5 mutant peptides, satisfying the criteria for T cell immune evasion. Only eleven mutations such as N211I, del212/212, V213E, L216F, I332V, G339H, N440K, K444T, V445P/H, G446S, and P681R were discovered to evade the CD8^+^ T cell response associated with BA.5 (Fig. 6F). The mutate sites that enhance the CD8^+^ T cell responses in Omicron variant spikes were summarized as Fig. 6G.

## Discussion

Under the selective pressure exerted by the immune system, SARS-CoV-2 continues to evolve, resulting in numerous mutations within its spike protein. This is particularly evident in the Omicron variants, which harbor a substantial number of mutations and has demonstrated an enhanced capacity to evade neutralizing antibodies during its transmission. However, the implications of these spike protein mutations on T cell-mediated immunity, specifically within the Omicron variants, remain incompletely understood. In this study, we successfully developed DNA vaccines encoding the spike protein of the PS as well as Omicron variants. These vaccines were emulsified with a Th1-type immune adjuvant, DM, and administered to BALB/c mice. Utilizing IFN-γ ELISPOT assays, we conducted a comprehensive analysis of the cross-reactive T cell responses targeting the spike proteins of these viral strains in the splenocytes of immunized mice. FACS was employed to identify peptide-specific IFN-γ^+^ CD4^+^ and IFN-γ^+^ CD8^+^ T cell responses within the splenocytes, providing landscapes of the peptide-specific T cell responses elicited by the spike proteins of different viral strains to elucidate the mechanisms underlying cross-T cell responses and the effects of mutations on T cell responses. Our findings significantly contribute to updating the emergency vaccine policies recommended by the WHO that are still in use in various countries, and provide key scientific basis for the design and development of more effective vaccines and immunization strategies to control the outbreak of emerging Omicron variant infections.

Previous studies have suggested that vaccines targeting the PS spike protein contain numerous conserved CD4^+^ and CD8^+^ T cell epitopes, as identified through epitope prediction, which may provide T cell-mediated immune protection against early viral variants [18]. However, our study is the first to reveal that only a limited number of mice and a small subset of splenocytes from mice immunized with the DNA vaccine expressing the PS spike protein were able to elicit cross-reactive T cell immune responses against the spike protein of Omicron variants, and these responses were notably weak. This phenomenon is further elucidated through a comparative landscape analysis of peptide-specific T cell responses, which underscores substantial differences in the T cell epitopes of the spike protein between the Omicron variants and the PS. Consequently, mutations significantly alter the T cell epitopes of the Omicron variant spikes. While our previous research utilized a 12-amino-acid (aa) peptide, the present study employed a 16-aa peptide to improve the recognition of B and T cell epitopes, resulting in a slightly altered landscape of peptide-specific T cell responses for the PS and Omicron BA.1 spike proteins. In previous studies, we determined that the T cell epitopes of the PS spike protein were primarily situated outside the N-terminus and RBD. Conversely, vaccination with the Omicron BA.1 variant spike demonstrated a concentrated T cell response to the PS’s peptides located at the C-terminus of the NTD and S2 regions, with minimal T cell response to the RBD peptides [18]. The findings of this study corroborate the earlier report. This investigation also revealed that the T cell epitopes of BA.1 are predominantly localized within its own regions, such as the NTD, RBD, and the central part of S2 of the spike protein. Mechanistically, it can be elucidated that, compared to the PS, at least 50 mutations in various Omicron variants facilitate the evasion of CD4^+^ T cell responses, while at least 37 mutations contribute to the evasion of CD8^+^ T cell responses. To date, some of these mutations have been identified within CD4^+^ and CD8^+^ T cell epitopes through functional experiments [31]. Notably, newly emerging mutations that impact T cell immune responses warrant further investigation. More importantly, this study’s findings suggest that emergency vaccines may not be effective in preventing severe disease and mortality caused by emerging Omicron variants. Real-world data further corroborate this conclusion. Specifically, the administration of two doses of the CoronaVac inactivated vaccine demonstrated an effectiveness of 90.3% in preventing severe illness and 86.3% in preventing mortality due to the earliest Omicron variant in individuals aged 16 and above [32]. However, the same vaccination regimen exhibited reduced effectiveness, with 77.8% in preventing severe illness and 82.7% in preventing mortality, against the Omicron BA.2 variant in individuals aged 18–50 [33]. Therefore, relying on the PS spike protein as a vaccine target antigen or on natural infection with early non-Omicron variants poses a significant risk of ineffectiveness in preventing severe disease and mortality associated with future Omicron variants.

Another important finding of this study is that DNA vaccines encoding the spike protein of predominant Omicron variants effectively elicited robust cross-reactive T cell immune responses in the splenocytes of immunized mice against diverse spike proteins from both PS and Omicron variants. Notably, DNA vaccines targeting the spike proteins of BA.1, BA.5, XBB.1.5, and JN.1 variants induced the most potent and broad cross-reactive T cell responses to other Omicron variants, attributed to the relatively low number of T cell immune evasion mutations in their spike proteins compared to BA.5. Furthermore, the distribution of T cell epitopes varies significantly among different Omicron variants, with these epitopes being dispersed across various structural domains of the Omicron variant’s spike protein. Importantly, the RBD regions of several Omicron variants consistently contain a substantial number of T cell epitopes, which partially explains the observed cross-reactivity of T cells among these variants. Previous studies have demonstrated that the BA.4/5 bivalent vaccine induces robust T cell responses against the spike proteins of Omicron BA.1, BA.2, and BA.5 variants [34], partially corroborating the results of this study. Compared to the BA.5 variant, other Omicron variants only possess several epitopes that facilitate T cell immune evasion, while simultaneously generating additional epitopes that enhance T cell responses. This phenomenon leads to substantial cross-reactivity of T cells among Omicron variants, although the mechanisms may be attributed to mutations in the spike protein of these viral strains that may alter its spatial conformation and thereby affecting the structure of epitopes [35, 36]. Previous researches hve demonstrated that vaccines employing the spike protein from BA.1, BA.5, and XBB variants as antigens can significantly reduce rates of COVID-19-related hospitalization [37, 38]. Collectively, these findings suggest that spike proteins from variants such as BA.1, BA.5, XBB.1.5, and JN.1 hold promise as target antigens for reducing the severity and mortality associated with diseases caused by Omicron variants. Considering that the JN.1 variant exhibits the strongest immune escape capabilities against neutralizing antibodies among Omicron sublineages in our recent study, the spike protein or RBD of the JN.1 variant is highly recommended as a vaccine target antigen.

In summary, our study demonstrated that cross-reactive T cell responses targeting the spike proteins of Omicron variants attribute to the mechanisms that the mutations on their T cell epitopes enhance T cell responses and only a few epitopes with mutations evade T cell responses. The PS spike protein specific cell-mediated immunity induced by vaccination or natural infection is ineffectiveness in preventing severe disease and mortality associated with emerging Omicron variants, due to weak cross-reactive T cell responses among the PS spike and Omicron counterparts, and at least 50 T cell evasion mutations in Omicron variant spike proteins. Our findings reveal that updating the emergency vaccine policies recommended by the WHO that are still in use in various countries, is both necessary and essential, and also benefit the design more effective vaccines for controlling the spread of infections and preventing the severity and death of diseases caused by the emergence of new Omicron variants.

## Conclusions

These findings underscore the imperative to update WHO emergency vaccine policies and contribute to the development of more effective vaccines and immunization strategies, to better control the infections caused by emerging Omicron variants.

## Supplementary Information

Below is the link to the electronic supplementary material.

Supplementary Material 1

## Data Availability

The datasets used and/or analysed during the current study are available from the corresponding author on reasonable request.

## Abbreviations

FACS: Flow cytometry
WHO: World health organization
China CDC: Chinese center for disease control and prevention
PS: Prototype strain
IFN-γ: Interferon-gamma
ELISPOT: Enzyme-linked immunospot assay
ICS: Intracellular cytokine staining
SPF: Specific-pathogen-free
SFC: Spot forming cells
NTD: N-terminal domain
RBD: Receptor-binding domain

## References

[1] Statement on the fifteenth meeting of the IHR (2005) Emergency Committee on the COVID-19 pandemic: https://www.who.int/news/item/05-05-2023-statement-on-the-fifteenth-meeting-of-the-international-health-regulations-(2005)-emergency-committee-regarding-the-coronavirus-disease-(covid-19)-pandemic.

[2] National Sentinel Surveillance Situation of Acute Respiratory Infectious Diseases (Week 23, 2025): https://www.chinacdc.cn/jksj/jksj04_14275/202506/t20250611_307561.html.

[3] Al KN, Zhang Y, Xia S, Yang Y, Al QM, Abdulrazzaq N, et al. Effect of 2 Inactivated SARS-CoV-2 Vaccines on Symptomatic COVID-19 Infection in Adults: A Randomized Clinical Trial. JAMA. 2021;326:35–45. 10.1001/jama.2021.8565. 34037666 10.1001/jama.2021.8565 PMC8156175

[4] Gray G, Collie S, Goga A, Garrett N, Champion J, Seocharan I, et al. Effectiveness of Ad26.COV2.S and BNT162b2 vaccines against Omicron variant in South Africa. N Engl J Med. 2022;386:2243–5. 10.1056/NEJMc2202061. 35507482 10.1056/NEJMc2202061 PMC9093716

[5] Castro DX, Ols S, Lore K, Karlsson HG. Immunity to SARS-CoV-2 induced by infection or vaccination. J Intern Med. 2022;291:32–50. 10.1111/joim.13372. 34352148 10.1111/joim.13372 PMC8447342

[6] Fujii SI, Yamasaki S, Iyoda T, Shimizu K. Association of cellular immunity with severity of COVID-19 from the perspective of antigen-specific memory T cell responses and cross-reactivity. Inflamm Regen. 2022;42:50. 10.1186/s41232-022-00239-1. 36447262 10.1186/s41232-022-00239-1 PMC9706959

[7] Cabezas C, Coma E, Mora-Fernandez N, Li X, Martinez-Marcos M, Fina F, et al. Associations of BNT162b2 vaccination with SARS-CoV-2 infection and hospital admission and death with covid-19 in nursing homes and healthcare workers in Catalonia: prospective cohort study. BMJ. 2021;374:n1868. 10.1136/bmj.n1868. 34407952 10.1136/bmj.n1868 PMC8371258

[8] Yan V, Wan E, Ye X, Mok A, Lai F, Chui C, et al. Effectiveness of BNT162b2 and CoronaVac vaccinations against mortality and severe complications after SARS-CoV-2 Omicron BA.2 infection: a case-control study. Emerg Microbes Infect. 2022;11:2304–14. 10.1080/22221751.2022.2114854. 35980089 10.1080/22221751.2022.2114854 PMC9553171

[9] Abdalla M, El-Arabey AA, Jiang X. Progress in research on the S protein as the target of COVID-19 vaccines. Expert Rev Vaccines. 2021;20:769–72. 10.1080/14760584.2021.1918003. 33853488 10.1080/14760584.2021.1918003

[10] Planas D, Saunders N, Maes P, Guivel-Benhassine F, Planchais C, Buchrieser J, et al. Considerable escape of SARS-CoV-2 Omicron to antibody neutralization. Nature. 2022;602:671–5. 10.1038/s41586-021-04389-z. 35016199 10.1038/s41586-021-04389-z

[11] Tan CW, Lim BL, Young BE, Yeoh AY, Yung CF, Yap WC, et al. Comparative neutralisation profile of SARS-CoV-2 omicron subvariants BA.2.75 and BA.5. Lancet Microbe. 2022;3:e898. 10.1016/S2666-5247(22)00220-8. 35963276 10.1016/S2666-5247(22)00220-8 PMC9365316

[12] Xi B, Zeng X, Chen Z, Zeng J, Huang L, Du H. SARS-CoV-2 within-host diversity of human hosts and its implications for viral immune evasion. MBio. 2023;14:e0067923. 10.1128/mbio.00679-23. 37273216 10.1128/mbio.00679-23 PMC10470530

[13] Zhang Y, Banga NJ, Gan M, Lin X, Fan X. Immune Evasive Effects of SARS-CoV-2 Variants to COVID-19 Emergency Used Vaccines. Front Immunol. 2021;12:771242. 10.3389/fimmu.2021.771242. 34880867 10.3389/fimmu.2021.771242 PMC8645832

[14] Tseng HF, Ackerson BK, Luo Y, Sy LS, Talarico CA, Tian Y, et al. Effectiveness of mRNA-1273 against SARS-CoV-2 Omicron and Delta variants. Nat Med. 2022;28:1063–71. 10.1038/s41591-022-01753-y. 35189624 10.1038/s41591-022-01753-y PMC9117141

[15] Zhu FC, Li YH, Guan XH, Hou LH, Wang WJ, Li JX, et al. Safety, tolerability, and immunogenicity of a recombinant adenovirus type-5 vectored COVID-19 vaccine: a dose-escalation, open-label, non-randomised, first-in-human trial. Lancet. 2020;395:1845–54. 10.1016/S0140-6736(20)31208-3. 32450106 10.1016/S0140-6736(20)31208-3 PMC7255193

[16] Tauzin A, Nayrac M, Benlarbi M, Gong SY, Gasser R, Beaudoin-Bussieres G, et al. A single dose of the SARS-CoV-2 vaccine BNT162b2 elicits Fc-mediated antibody effector functions and T cell responses. Cell Host Microbe. 2021;29:1137–50. 10.1016/j.chom.2021.06.001. 34133950 10.1016/j.chom.2021.06.001 PMC8175625

[17] Wang H, Gan M, Wu B, Zeng R, Wang Z, Xu J, et al. Humoral and cellular immunity of two-dose inactivated COVID-19 vaccination in Chinese children: a prospective cohort study. J Med Virol. 2023;95:e28380. 10.1002/jmv.28380. 36478357 10.1002/jmv.28380 PMC9877748

[18] Tarke A, Coelho CH, Zhang Z, Dan JM, Yu ED, Methot N, et al. SARS-CoV-2 vaccination induces immunological T cell memory able to cross-recognize variants from Alpha to Omicron. Cell. 2022;185:847–59. 10.1016/j.cell.2022.01.015. 35139340 10.1016/j.cell.2022.01.015 PMC8784649

[19] Gao Y, Cai C, Grifoni A, Muller TR, Niessl J, Olofsson A, et al. Ancestral SARS-CoV-2-specific T cells cross-recognize the Omicron variant. Nat Med. 2022;28:472–6. 10.1038/s41591-022-01700-x. 35042228 10.1038/s41591-022-01700-x PMC8938268

[20] Tarke A, Sidney J, Methot N, Yu ED, Zhang Y, Dan JM, et al. Impact of SARS-CoV-2 variants on the total CD4(+) and CD8(+) T cell reactivity in infected or vaccinated individuals. Cell Rep Med. 2021;2:100355. 10.1016/j.xcrm.2021.100355. 34230917 10.1016/j.xcrm.2021.100355 PMC8249675

[21] Polack FP, Thomas SJ, Kitchin N, Absalon J, Gurtman A, Lockhart S, et al. Safety and Efficacy of the BNT162b2 mRNA Covid-19 Vaccine. N Engl J Med. 2020;383:2603–15. 10.1056/NEJMoa2034577. 33301246 10.1056/NEJMoa2034577 PMC7745181

[22] June CY, Yi S, Hwang I, Kim J, Park YJ, Cho E, et al. Safety and effectiveness of BNT162b2 mRNA Covid-19 vaccine in adolescents. Vaccine. 2022;40:691–4. 10.1016/j.vaccine.2021.12.044. 35012777 10.1016/j.vaccine.2021.12.044 PMC8702409

[23] Tye E, Jinks E, Haigh TA, Kaul B, Patel P, Parry HM, et al. Mutations in SARS-CoV-2 spike protein impair epitope-specific CD4(+) T cell recognition. Nat Immunol. 2022;23:1726–34. 10.1038/s41590-022-01351-7. 36456735 10.1038/s41590-022-01351-7

[24] Impaired CD4(+) T cell recognition of SARS-CoV-2 variants of concern. Nat Immunol. 2022; 23:1671–1672. 10.1038/s41590-022-01353-5. 10.1038/s41590-022-01353-5 PMC9734360 36474117

[25] Agerer B, Koblischke M, Gudipati V, Montano-Gutierrez LF, Smyth M, Popa A, et al. SARS-CoV-2 mutations in MHC-I-restricted epitopes evade CD8(+) T cell responses. Sci Immunol. 2021. 10.1126/sciimmunol.abg6461. 33664060 10.1126/sciimmunol.abg6461 PMC8224398

[26] Emmelot ME, Vos M, Boer MC, Rots NY, de Wit J, van Els C, et al. Mutations in SARS-CoV-2 Spike Lead to Reduced T-Cell Response in Vaccinated and Convalescent Individuals. Viruses. 2022. 10.3390/v14071570. 35891550 10.3390/v14071570 PMC9318964

[27] Emmelot ME, Vos M, Boer MC, Rots NY, van Els C, Kaaijk P. SARS-CoV-2 Omicron BA.4/BA.5 Mutations in Spike Leading to T Cell Escape in Recently Vaccinated Individuals. Viruses. 2022. 10.3390/v15010101. 36680141 10.3390/v15010101 PMC9863717

[28] Gan M, Cao J, Zhang Y, Fu H, Lin X, Ouyang Q, et al. Landscape of T cell epitopes displays hot mutations of SARS-CoV-2 variant spikes evading cellular immunity. J Med Virol. 2024;96:e29452. 10.1002/jmv.29452. 38314852 10.1002/jmv.29452

[29] Zhang H, Deng S, Ren L, Zheng P, Hu X, Jin T, et al. Profiling CD8(+) T cell epitopes of COVID-19 convalescents reveals reduced cellular immune responses to SARS-CoV-2 variants. Cell Rep. 2021;36:109708. 10.1016/j.celrep.2021.109708. 34506741 10.1016/j.celrep.2021.109708 PMC8390359

[30] Hao L, Wu Y, Zhang Y, Zhou Z, Lei Q, Ullah N, et al. Combinational PRR Agonists in Liposomal Adjuvant Enhances Immunogenicity and Protective Efficacy in a Tuberculosis Subunit Vaccine. Front Immunol. 2020;11:575504. 10.3389/fimmu.2020.575504. 33117374 10.3389/fimmu.2020.575504 PMC7561437

[31] Jin X, Liu X, Shen C. A systemic review of T-cell epitopes defined from the proteome of SARS-CoV-2. Virus Res. 2023;324:199024. 10.1016/j.virusres.2022.199024. 36526016 10.1016/j.virusres.2022.199024 PMC9757803

[32] Jara A, Undurraga EA, Gonzalez C, Paredes F, Fontecilla T, Jara G, et al. Effectiveness of an inactivated SARS-CoV-2 vaccine in Chile. N Engl J Med. 2021;385:875–84. 10.1056/NEJMoa2107715. 34233097 10.1056/NEJMoa2107715 PMC8279092

[33] Urschel R, Bronder S, Klemis V, Marx S, Hielscher F, Abu-Omar A, et al. SARS-CoV-2-specific cellular and humoral immunity after bivalent BA.4/5 COVID-19-vaccination in previously infected and non-infected individuals. Nat Commun. 2024;15:3077. 10.1038/s41467-024-47429-8. 38594497 10.1038/s41467-024-47429-8 PMC11004149

[34] Starr TN, Greaney AJ, Hilton SK, Ellis D, Crawford K, Dingens AS, et al. Deep Mutational Scanning of SARS-CoV-2 Receptor Binding Domain Reveals Constraints on Folding and ACE2 Binding. Cell. 2020;182:1295–310. 10.1016/j.cell.2020.08.012. 32841599 10.1016/j.cell.2020.08.012 PMC7418704

[35] Barnes CO, West AJ, Huey-Tubman KE, Hoffmann M, Sharaf NG, Hoffman PR, et al. Structures of Human Antibodies Bound to SARS-CoV-2 Spike Reveal Common Epitopes and Recurrent Features of Antibodies. Cell. 2020;182:828–42. 10.1016/j.cell.2020.06.025. 32645326 10.1016/j.cell.2020.06.025 PMC7311918

[36] Hansen CH, Moustsen-Helms IR, Rasmussen M, Soborg B, Ullum H, Valentiner-Branth P. Short-term effectiveness of the XBB.1.5 updated COVID-19 vaccine against hospitalisation in Denmark: a national cohort study. Lancet Infect Dis. 2024;24:e73–4. 10.1016/S1473-3099(23)00746-6. 38190834 10.1016/S1473-3099(23)00746-6

[37] Lee IT, Cosgrove CA, Moore P, Bethune C, Nally R, Bula M, et al. Omicron BA.1-containing mRNA-1273 boosters compared with the original COVID-19 vaccine in the UK: a randomised, observer-blind, active-controlled trial. Lancet Infect Dis. 2023;23:1007–19. 10.1016/S1473-3099(23)00295-5. 37348519 10.1016/S1473-3099(23)00295-5

[38] Tenforde MW, Weber ZA, Natarajan K, Klein NP, Kharbanda AB, Stenehjem E, et al. Early estimates of bivalent mRNA vaccine effectiveness in preventing COVID-19-associated emergency department or urgent care encounters and hospitalizations among immunocompetent adults - VISION network, nine states, September-November 2022. MMWR Morb Mortal Wkly Rep. 2023;71:1637–46. 10.15585/mmwr.mm7153a1. 36921274 10.15585/mmwr.mm7153a1 PMC10027383

