# Supplementary material for: Extensive cross-reactive T cell epitopes across SARS-CoV-2 Omicron variant spikes with finite immune evasion mutations
Source: J Transl Med. 2025 Sep 30;23:1027. doi: 10.1186/s12967-025-07076-z (PMC12487199; doi:10.1186/s12967-025-07076-z)
Supplement: Supplementary file 1 — Supplementary Material 1 [file 12967_2025_7076_MOESM1_ESM.pdf]

**Supplementary Materials for**

# Extensive cross-reactive T cell epitopes across SARS-CoV-2 Omicron variant spikes with finite immune evasion mutations

**This PDF file includes:**

**Figure S1**

**Table S1**

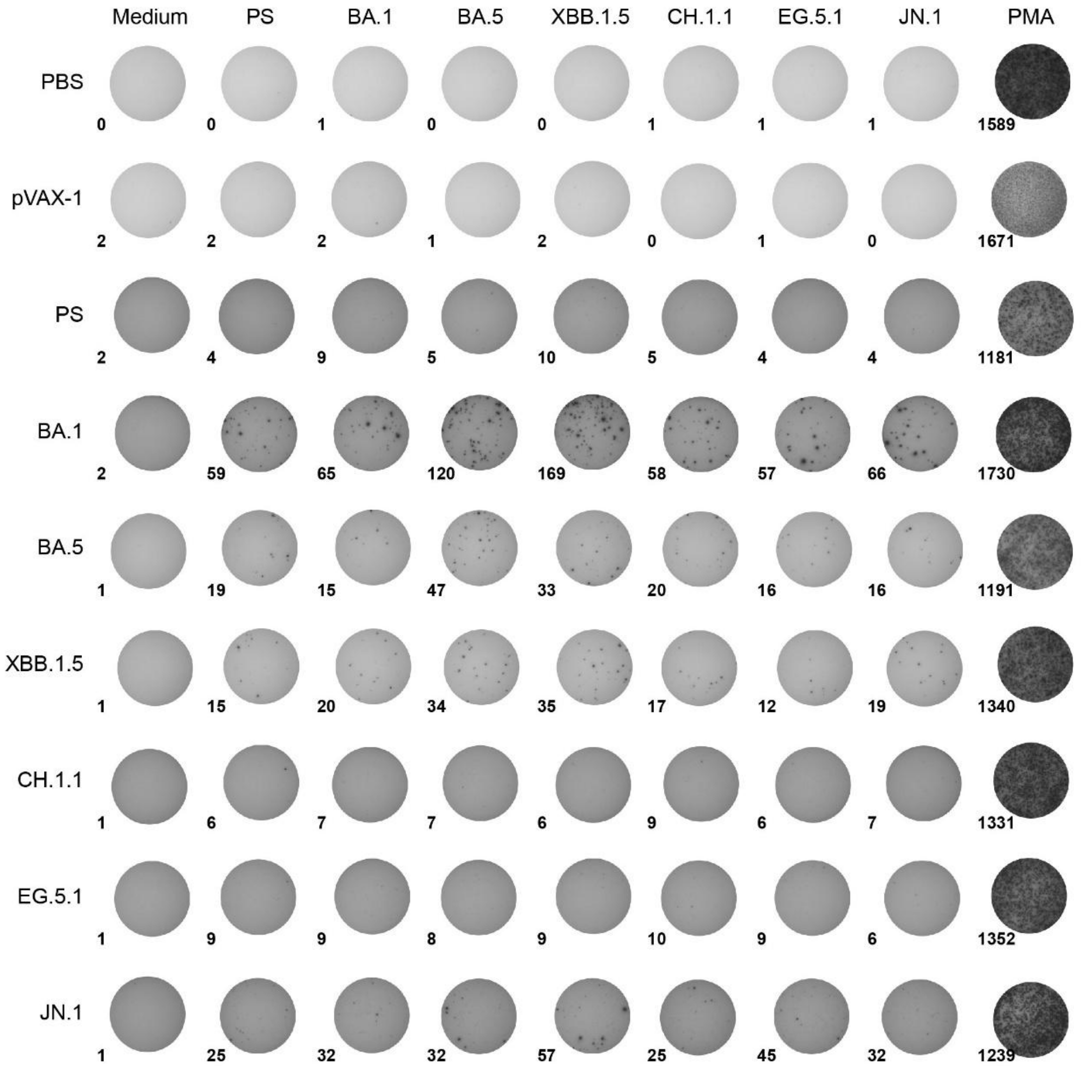


**Figure S1. ELISPOT Analysis of Spike Protein-Specific T Cell Responses to SARS-CoV-2 PS and Variant Strains**

**Supplementary Table S1.** Mutant peptides, mutation site and sequences

| Peptide name | Mutation sites | Peptide sequence |
|---|---|---|
| O4-1 | V3G | MFGFLVLLPLVSSQCV |
| O#-2 | T19I,L24S | PLVSSQCVNLITRTQS |
| JN-2 | T19I,R21T,L24S | PLVSSQCVNLITTTQS |
| O#-3 | T19I,L24S,del25/27 | NLITRTQS---YTNSF |
| JN-3 | T19I,R21T,L24S,del25/27 | NLITTTQS---YTNSF |
| O*-4 | del25/27 | _--YTNSFTRGVYYPD |
| EV-6 | Q52H | KVFRSSVLHSTHDLFL |
| JN-6 | S50L | KVFRSSVLHLTQDLFL |
| EV-7 | Q52H | HSTHDLFLPFFSNVTW |
| JN-7 | S50L | HLTQDLFLPFFSNVTW |
| O1-8 | A67V,del69/70 | PFFSNVTWFHVI--SG |
| AO2457QJ-8 | del69/70 | PFFSNVTWFHAI--SG |
| O1-9 | A67V,del69/70 | FHVI--SGTNGTKRFD |
| AO2457QJ-9 | del69/70 | FHAI--SGTNGTKRFD |
| XEV-10 | V83A | TNGTKRFDNPALPFND |
| O1-11 | T95I | NPVLPFNDGVYFASIE |
| XEV-11 | V83A | NPALPFNDGVYFASTE |
| O1-12 | T95I | GVYFASIEKSNIIRGW |
| JN-15 | V127F | KTQSLLIVNNATNVFI |
| JN-16 | V127F | NNATNVFIKVCEFQFC |

| | | |
|---|---|---|
| O1-17 | G142D,del143/145 | KVCEFQFCNDPFLD-- |
| O*-17 | G142D | KVCEFQFCNDPFLDVY |
| O1-18 | G142D,del143/145 | NDPFLD---HKNNKSW |
| O2457-18 | G142D | NDPFLDVYYHKNNKSW |
| XEV-18 | G142D,del144/144,H146Q | NDPFLDV-YQKNNKSW |
| CH-18 | G142D,K147E,W152R | NDPFLDVYYHENNKSR |
| QJ-18 | G142D,del144/144 | NDPFLDVY-HKNNKSW |
| AO1Q-19 | del143/145 | _HKNNKSWMESEFRVY |
| CH-19 | K147E,W152R,F157L | YHENNKSRMESELRVY |
| XE-19 | del144/144,H146Q | _QKNNKSWMESEFRVY |
| HV-19 | del144/144,H146Q,F157L | _QKNNKSWMESELRVY |
| JN-19 | del144/144,F157S,R158G | —HKNNKSWMESESGVY |
| CV-20 | F157L | MESELRVYSSANNCTF |
| JN-20 | F157S,R158G | MESESGVYSSANNCTF |
| XEV-22 | Q183E | EYVSQPFLMDLEGKEG |
| XEV-23 | Q183E | MDLEGKEGNFKNLREF |
| O1-26 | N211I,del212/212 | FKIYSKHTPII-VRDL |
| O2457Q-26 | V213G | FKIYSKHTPINLGRDL |
| CH-26 | I210V, V213G | FKIYSKHTPVNLGRDL |
| XEV-26 | Q183E | FKIYSKHTPINLERDL |
| JN-26 | N211I,del212/212,V213G,L216F | FKIYSKHTPI-IGRDF |
| O1-27 | N211I,del212/212 | PII-VRDLPQGFSALE |

| | | |
|---|---|---|
| O2457-27 | V213G | PINLGRDLPQGFSALE |
| CH-27 | I210V, V213G | PVNLGRDLPQGFSALE |
| XEV-27 | Q183E | PINLERDLPQGFSALE |
| JN-27 | N211I,del212/212,V213G,L216F | PI-IGRDFPQGFSALE |
| JN-30 | H245N | INITRFQTLLALNRSY |
| XEV-31 | G252V | LLALHRSYLTPVDSSS |
| JN-31 | H245N | LLALNRSYLTPGDSSS |
| XEV-32 | G252V | LTPVDSSSGWTAGAAA |
| CH-32 | G257S | LTPGDSSSSWTAGAAA |
| JN-32 | A264D | LTPGDSSSGWTAGAAD |
| CH-33 | G257S | SWTAGAAAYYVGYLQP |
| JN-33 | A264D | GWTAGAADYYVGYLQP |
| JN-41 | I332V | QPTESIVRFPNVTNLC |
| O12457Q-42 | G339D | FPNITNLCPFDEVFNA |
| XCEV-42 | G339H | FPNITNLCPFHEVFNA |
| JN-42 | I332V,G339H | FPNVTNLCPFHEVFNA |
| O1-43 | G339D,R346K | PFDEVFNATKFASVYA |
| O245-43 | G339D | PFDEVFNATRFASVYA |
| O7Q-43 | G339D,R346T | PFDEVFNATTFASVYA |
| XCEV-43 | G339H | PFHEVFNATTFASVYA |
| JN-43 | G339H | PFHEVFNATRFASVYA |
| O1-44 | R346K | TKFASVYAWNRKRISN |

| | | |
|---|---|---|
| O7XQCEV-44 | R346T | TTFASVYAWNRKRISN |
| JN-44 | K356T | TRFASVYAWNRTRISN |
| XEV-45 | L368I | WNRKRISNCVADYSVI |
| JN-45 | K356T | WNRTRISNCVADYSVL |
| O1-46 | S371L,S373P,S375F | CVADYSVLYNLAPFFT |
| O2457QCJ-46 | S371L,S373P,S375F,T376A | CVADYSVLYNFAPFFA |
| XEV-46 | L368I,S371F,S373P,S375F,T376A | CVADYSVIYNFAPFFA |
| O1-47 | S371L,S373P,S375F | YNLAPFFTFKCYGVSP |
| O*-47 | S371L,S373P,S375F,T376A | YNFAPFFAFKCYGVSP |
| O#-50 | D405N,R408S | TNVYADSFVIRGNEVS |
| JN-50 | R403K,D405N,R408S | TNVYADSFVIKGNEVS |
| O#-51 | D405N,R408S | VIRGNEVSQIAPGQTG |
| JN-51 | R403K,D405N,R408S | VIKGNEVSQIAPGQTG |
| BO-52 | K417N | QIAPGQTGNIADYNYK |
| BO-53 | K417N | NIADYNYKLPDDFTGC |
| O*-54 | N440K | LPDDFTGCVIAWNSNK |
| O1-55 | G446S | VIAWNSNNLDSKVSGN |
| O2457-55 | N440K | VIAWNSNKLDSKVGGN |
| XEV-55 | N440K,V445P,G446S | VIAWNSNKLDSKPSGN |
| OQ-55 | N440K,K444T | VIAWNSNKLDSTVGGN |
| CH-55 | N440K,K444T,G446S | VIAWNSNKLDSTVSGN |
| JN-55 | N440K,V445H,G446S | VIAWNSNKLDSKHSGN |

| | | |
|---|---|---|
| O1-56 | G446S | LDSKVSGNYNYLYRLF |
| DO2457-56 | L452R | LDSKVGGNYNYRYRLF |
| OX-56 | V445P,G446S | LDSKPSGNYNYLYRLF |
| OQ-56 | K444T,L452R | LDSTVGGNYNYRYRLF |
| CH-56 | K444T,G446S,L452R | LDSTVSGNYNYRYRLF |
| EG-56 | V445P,G446S,F456L | LDSKPSGNYNYLYRLL |
| HV-56 | V445P,G446S,L452R,F456L | LDSKPSGNYNYRYRLL |
| JN-56 | V445H,G446S,N450D,L452W,L455S | LDSKHSGNYDYWYRSF |
| DO2457-57 | L452R | YNYRYRLFRKSNLKPF |
| OX-57 | N460K | YNYLYRLFRKSKLKPF |
| OQC-57 | L452R, N460K | YNYRYRLFRKSKLKPF |
| EG-57 | F456L,N460K | YNYLYRLLRKSKLKPF |
| HV-57 | L452R,F456L,N460K | YNYRYRLLRKSKLKPF |
| JN-57 | N450D,L452W,L455S,N460K | YDYWYRSFRKSKLKPF |
| OXQCEVJ-58 | N460K | RKSKLKPFERDISTEI |
| O-59 | S477N,T478K | ERDISTEIYQAGNKPC |
| O12-60 | S477N,T478K,E484A | YQAGNKPCNGVAGFNC |
| O457Q-60 | S477N,T478K,E484A,F486V | YQAGNKPCNGVAGVNC |
| CH-60 | S477N,T478K,E484A,F486S | YQAGNKPCNGVAGSNC |
| XEV-60 | S477N,T478K,E484A,F486P | YQAGNKPCNGVAGPNC |
| JN-60 | S477N,T478K,N481K,del483/483,E484K,F486P | YQAGNKPCKG-KGPNC |
| O1-61 | E484A,Q493R,G496S | NGVAGFNCYFPLRSYS |

| | | |
|---|---|---|
| O2-61 | E484A,Q493R | NGVAGFNCYFPLRSYG |
| O457Q-61 | E484A,F486V | NGVAGVNCYFPLQSYG |
| CH-61 | E484A,F486S | NGVAGSNCYFPLQSYG |
| XEV-61 | E484A,F486P,F490S | NGVAGPNCYSPLQSYG |
| JN-61 | N481K,del483/483,E484K,F486P | KG-KGPNCYFPLQSYG |
| O1-62 | Q493R,G496S,Q498R,N501Y | YFPLRSYSFRPTYGVG |
| O2-62 | Q493R,Q498R,N501Y | YFPLRSYGFRPTYGVG |
| O457QCJ-62 | Q498R,N501Y | YFPLQSYGFRPTYGVG |
| XEV-62 | F490S,Q498R,N501Y | YSPLQSYGFRPTYGVG |
| O-63 | Q498R,N501Y,Y505H | FRPTYGVGHQPYRVVV |
| O-64 | Y505H | HQPYRVVVLSFELLHA |
| O1-68 | T547K | KCVNFNFNGLKGTGVL |
| O1-69 | T547K | GLKGTGVLTESNKKFL |
| JN-69 | E554K | GLTGTGVLTKSNKKFL |
| JN-70 | E554K | TKSNKKFLPFQQFGRD |
| JN-71 | A570V | PFQQFGRDIVDTTDAV |
| JN-72 | A570V | IVDTTDAVRDPQTLEI |
| ABGDO-76 | D614G | GTNTSNQVAVLYQGVN |
| ABGDO&-77 | D614G | AVLYQGVNCTEVPVAI |
| JN-77 | D614G,P621S | AVLYQGVNCTEVSVAI |
| JN-78 | P621S | CTEVSVAIHADQLTPT |
| GO-81 | H655Y | NVFQTRAGCLIGAEYV |

| | | |
|---|---|---|
| GO-82 | H655Y | CLIGAEYVNNSYECDI |
| O-84 | N679K | PIGAGICASYQTQTKS |
| O&-85 | N679K,P681H | SYQTQTKSHRRARSVA |
| JN-85 | N679K,P681R | SYQTQTKSRRRARSVA |
| AO&-86 | P681H | HRRARSVASQSIIAYT |
| DJ-86 | P681R | RRRARSVASQSIIAYT |
| O-95 | N764K | LLQYGSFCTQLKRALT |
| O-96 | N764K | TQLKRALTGIAVEQDK |
| O-99 | D796Y | VKQIYKTPPIKYFGGF |
| O-100 | D796Y | PIKYFGGFNFSQILPD |
| O1-106 | N856K | LGDIAARDLICAQKFK |
| O1-107 | N856K | LICAQKFKGLTVLPPL |
| JN-117 | S939F | SAIGKIQDSLFSTASA |
| JN-118 | S939F | SLFSTASALGKLQDVV |
| O-119 | Q954H | LGKLQDVVNHNAQALN |
| O-120 | Q954H | NHNAQALNTLVKQLSS |
| O-121 | N969K | TLVKQLSSKFGAISSV |
| O1-122 | N969K,L981F | KFGAISSVLNDIFSRL |
| O*-122 | N969K | KFGAISSVLNDILSRL |
| JN-142 | P1143L | VIGIVNNTVYDPLQLE |
| JN-143 | P1143L | VYDPLQLELDSFKEEL |

Peptide name abbreviation: A: Alpha. B: Beta. G: Gamma. D: Delta. O: Omicron. AO457: There

are mutations in the Alpha, Omicron BA.4, BA.5, BF.7. ABGDO: It means that there are mutations in all the variant strains. O1: Omicron BA.1. O4: Omicron BA.4. XCEV: XBB.1.5, CH.1.1, EG.5.1.1, HV.1. O# indicates all variants of Omicron except BA.1 and JN.1. O* indicates all variants of Omicron except BA.1. O& indicates all variants of Omicron except JN.1. - Indicates deleted.
